# Checkpoint kinase interaction with DNA polymerase alpha regulates replication progression during stress

**DOI:** 10.12688/wellcomeopenres.19617.1

**Published:** 2023-07-26

**Authors:** Andreas Hadjicharalambous, Alex J. Whale, Geylani Can, J. Mark Skehel, Jonathan M. Houseley, Philip Zegerman

**Affiliations:** 1 Department of Biochemistry, University of Cambridge, Cambridge, England, CB2 1GA, UK; 2 Epigenetics Programme, Babraham Institute, University of Cambridge, Cambridge, England, CB22 3AT, UK; 3 Medical Research Council Laboratory of Molecular Biology, Cambridge Biomedical Campus, London, England, CB2 0QH, UK

**Keywords:** DNA replication, cell cycle, checkpoint, genome stability, cancer

## Abstract

**Background:** In eukaryotes, replication stress activates a checkpoint response, which facilitates genome duplication by stabilising the replisome. How the checkpoint kinases regulate the replisome remains poorly understood. The aim of this study is to identify new targets of checkpoint kinases within the replisome during replication stress.

**Methods:** Here we use an unbiased biotin proximity-ligation approach in *Saccharomyces cerevisiae* to identify new interactors and substrates of the checkpoint kinase Rad53 *in vivo.*

**Results:** From this screen, we identified the replication initiation factor Sld7 as a Rad53 substrate, and Pol1, the catalytic subunit of polymerase a, as a Rad53-interactor. We showed that CDK phosphorylation of Pol1 mediates its interaction with Rad53. Combined with other interactions between Rad53 and the replisome, this Rad53-Pol1 interaction is important for viability and replisome progression during replication stress.

**Conclusions:** Together, we explain how the interactions of Rad53 with the replisome are controlled by both replication stress and the cell cycle, and why these interactions might be important for coordinating the stabilisation of both the leading and lagging strand machineries.

## Introduction

Genome instability resulting from defective DNA replication and repair is a hallmark of the early stages of oncogenesis ( Kotsantis *et al.*, 2018). In normal dividing cells, genome duplication is tightly regulated and strictly monitored to ensure that every chromosome is replicated in its entirety. For eukaryotes, this regulation includes coupling the two steps in replication—licensing and initiation—to the cell cycle, a plethora of repair pathways that act during and after replication, and a system of checkpoint kinases that respond to perturbed replication by coordinating repair and impeding cell cycle progression. Defects in any of these processes are associated with tumour development ( Abbas *et al.*, 2013), but are also potential vulnerabilities that can be exploited to specifically kill cancer cells ( da Costa *et al.*, 2023).

During genome duplication, DNA lesions or low levels of deoxynucleotide triphosphates (dNTPs) cause stalling of the replisome, leading to the exposure of single stranded DNA at the replication fork ( Saldivar *et al.*, 2017). This stalling is a key trigger for activation of the apical checkpoint kinase called ATR in humans and Mec1 in budding yeast. In conjunction with a mediator protein (Claspin/Mrc1) that binds to the replisome ( Errico & Costanzo, 2012), ATR/Mec1 activation subsequently leads to the activation of the effector kinase Chk1 in humans or Rad53 in yeast ( Giannattasio & Branzei, 2017). This response is called the S-phase, intra-S-phase or DNA replication checkpoint ( Pardo *et al.*, 2017).

The S-phase checkpoint responds to replication defects by upregulating dNTPs, coordinating DNA repair and by inhibiting further origin firing ( Giannattasio & Branzei, 2017; McClure *et al.*, 2022; Saldivar *et al.*, 2017). A critical function of the checkpoint response to stalled DNA replication is also to allow the resumption of genome duplication after stalling, in a process called fork stabilisation ( Giannattasio & Branzei, 2017; McClure *et al.*, 2022; Saldivar *et al.*, 2017). In cells that lack checkpoint activity, replication forks cannot continue DNA synthesis after stalling ( McClure *et al.*, 2022), DNA unwinding and synthesis become uncoupled ( Gan *et al.*, 2017) and the fork is said to have ‘collapsed’ ( Giannattasio & Branzei, 2017; McClure *et al.*, 2022; Saldivar *et al.*, 2017). How the checkpoint kinases prevent fork collapse is poorly understood, but the replisome itself remains largely intact even in the absence of checkpoint kinases ( De Piccoli *et al.*, 2012; Dungrawala *et al.*, 2015).

Several targets of the checkpoint kinases have been identified within the replisome, demonstrating that these kinases regulate multiple processes including fork rate, exonuclease activity and fork remodelling ( Giannattasio & Branzei, 2017; McClure *et al.*, 2022; Pellicano *et al.*, 2021; Saldivar *et al.*, 2017). Identifying additional functions of the checkpoint kinases in replisome stabilisation is complicated by the fact that the checkpoint kinases have very low substrate specificities ( Blasius *et al.*, 2011; Mok *et al.*, 2010) and phosphorylate proteins at multiple sites ( Zegerman & Diffley, 2010), making it difficult to probe the function of specific phosphorylation events. In yeast, we and others have shown that Rad53 substrate specificity can be mediated by protein-protein interactions ( Can *et al.*, 2019; Chen *et al.*, 2013; Smolka *et al.*, 2006). For example, Rad53 binds to the initiation factor Cdc45, which targets Rad53 to Sld3 to inhibit origin firing and also recruits Rad53 to the replisome ( Can *et al.*, 2019). Rad53 also directly binds to substrates such as Dbf4 ( Chen *et al.*, 2013). From this, we reasoned that identifying the interactions of the checkpoint kinases with the replisome *in vivo* might be a promising approach to determine novel functions of these kinases at the fork.

Here we utilise an unbiased proximity-dependent biotin identification (BioID) screening approach in the budding yeast *Saccharomyces cerevisiae* to identify new replication partners of the effector checkpoint kinase Rad53 *in vivo*. Using this method, we identify new Rad53 substrates including the replication initiation factor Sld7, and we pinpoint the catalytic subunit of polymerase α (Pol1) as a new binding partner for Rad53 in the replisome. Together with interactions of Rad53 with the leading strand machinery, this study provides insight into the multitude of binding sites for Rad53 within the replisome that are important for the progression of stalled replication complexes *in vivo*.

## Methods

### Yeast strains

Yeast strains can be found in Supplementary Table 2 as *Extended data* ( Zegerman, 2023).

### Yeast transformation

Yeast was grown to exponential phase at a concentration of 10 ^7^ cells/ml. For each transformation, 10 ml of culture was spun down and washed with distilled water. They were resuspended with 1 ml of solution 1 (0.1 M lithium acetate and 1xTE, both at pH 7.5), spun down and 0.95 ml of supernatant was removed. In total, 5 μl of ssDNA (10 mg/ml) and DNA to be inserted was then added (100–400 ng of DNA/transformation). Then, 300 μl of solution 2 (40% PEG 4000, 0.1 M lithium acetate and 1xTE, both at pH 7.5) was used to resuspend the culture. The mixture was incubated at 30°C for 30 minutes and then DMSO was added to make a final 10% of the mixture. The solution was then heat-shocked for 15 minutes at 42°C and then rapidly cooled for 2 minutes on ice. Cells were spun and either resuspended in 100 μl of distilled water (for constructs containing amino acid or nucleotide markers) or in 1 ml of YPD and incubated at 30°C for 3 hours (for constructs with drug resistance markers). The cells were then plated on selective plates and incubated for 36–48 hours at 30°C.

### Yeast genomic DNA (gDNA) extraction

A total of 10 ml of yeast culture was grown overnight. Cells were pelleted and resuspended in 200 μl of lysis buffer (100 mM NaCl, 10 mM Tris pH 8.0, 1 mM EDTA, 1% SDS, 2% Triton X-100). In total, 200 μl of glass beads, 0.45 mm diameter, and 200 μl of Phenol Chloroform pH 8.0 was added. The mixture was vortexed for 30 seconds, 200 μl of TE buffer (10 mM Tris pH 8, 1 mM EDTA pH 8.0) was added and revortexed for another 10 seconds. The mixture was spun at 13 krpm on a benchtop centrifuge for 2 minutes. Then, 380 μl of supernatant was transferred to a fresh Eppendorf and 760 μl of 100% ethanol was added. The solution was spun at the same conditions as before to precipitate the DNA. A total of 1 ml of 70% ethanol was added (to wash the salts from the pellet) and the solution was re-spun as before. The pellet was air dried, resuspended in 50 μl of TE with RNAse (1 μg/ml) and incubated at 37°C for 1 hour.

### Yeast timecourse experiments

Yeast cultures were grown overnight in YPD or SCD media to exponential phase. Cultures were made to a concentration of 10 ^7 ^cells/ml and then were arrested in G1 (with 5 μg/ml of alpha factor) or G2/M (with 10 μg/ml nocodazole) for 90 minutes. For G1 block and release, cells were pelleted and washed twice with YPD/SCD media and then released into the equivalent media with 200 mM HU, 0.01% MMS or no drug at 30°C with agitation. For nocodazole blocked experiments, cells were incubated with 5 μg/ml of phleomycin and 10 μg/ml of nocodazole. Cells were removed every 20 or 30 minutes for FACS analysis (500 μl, pelleted and fixed in 300 μl of 70% ethanol), for TCA sample preparation (5 ml, pelleted and flash frozen in dry ice) or for budding index analysis (500 μl sonicated and put on a glass slide for optical microscopy).

### Yeast whole cell lysate preparation using trichloroacetic acid (TCA extracts)

A total of 5 ml of yeast culture with density of 10 ^7^ cells/ml was pelleted, and the pellet resuspended in 200 μl of 20%(w/v) TCA. Solution was transferred in a screw-caped tube with 200 μl of glass beads (0.45 mm diameter) and then shaken on a bead beater (Precellys 24 Homogenizer) twice at 5,000 speed for 30 seconds with a 45 second break in between the shakes. 5% (w/v) 400 μl TCA was then added to and the liquid was transferred in a new tube. This was repeated twice to wash as much of the protein from the beads as possible. The solution was then spun at 13 krpm on a benchtop centrifuge for 2 minutes. The supernatant was removed and the pellet was first resuspended in 250 μl of loading buffer (200 mM Tris pH 9.0, 100 mM Tris pH 6.8, 0.8% SDS, 16% glycerol, 1% (w/v) bromophenol blue, 8% β-mercaptoethanol) and boiled at 99°C for 10 minutes. The solution was then spun down for 2 minutes at 13.3 krpm and either stored at -20°C or used as a protein sample for SDS-PAGE.

### Yeast growth assays

Cell cultures were grown overnight to exponential phase and diluted to 10 ^7^ cells/ml. Using a 96-well plate, each strain was serially diluted by a factor of 3. A sterile replica plater and freshly made, dried agar plates were used to imprint the plates. After culture spots dried on the plates, they were incubated for 1–4 days at 30°C unless otherwise stated. Plates were scanned every 24 hours. Concentrations of drugs used: Hydroxyurea: 25 mM, 50 mM, 100 mM or 200 mM; MMS: 0.0025%, 0.005% or 0.01%, Camptothecin (CPT): 20 μM; NQO: 20 ng/ml; Phleomycin 1.5 μg/ml. Using ImageJ 1.53K (RRID:SCR_003070), the average mean grey value of the 3 ^rd^ dilution for each strain over 4 days was calculated for three biological repeats. The average and standard deviation was used to generate a graph of growth rates.

### Phos-tag western blots

Phos-tag gels were run using the Biorad mini-PROTEAN gel tank system. The resolving gel was 4% and was made using 424 μl 40% acrylamide, 292 μl 2% Bis-acrylamide, 1,875 μl Tris-HCl pH 8.8, 20 μl Phos-tag Acrylamide, 20 μl 10 mM MnCl _2_, 25 μl 20% SDS, 622 μl, 1,667 μl 1.5% agarose, 5 μl TEMED and 25 μl 10% APS. When solidified, the upper agarose layer was removed and stacking gel was added. After gel polymerisation, 60–80 μg of protein sample was loaded per lane, together with 6 μl of Amersham™ Rainbow Marker per experiment. Empty lanes were loaded with 5 μl of 1x Laemmli buffer (62.5 mM Tris pH 6.8, 0.5% SDS, 10% glycerol, bromophenol blue, 5% β-mercaptoethanol). The gel was run in 1L 1 x SDS buffer (10 L stock; 5x): 150 g Tris base, 720 g Glycine, 250 ml 20% SDS) at 20 mA / gel for 2–3 hours. Afterwards, the gel was removed from the glass container and washed in 0.02 M EDTA for 10 minutes to remove the manganese ions. This was repeated thrice and then the gel was washed in 1x wet transfer buffer (48 mM Tris base, 30 mM Glycine, 0.0375% SDS, 20% methanol).

After SDS-PAGE, proteins were transferred onto Amersham Protran™ 0.45 μm nitrocellulose membrane. The gel-membrane layer was covered on either side with filter paper. If the target protein was of an apparent molecular weight of >130 kDa, then wet transfer was used (Hoefer TE62 Standard Transfer Tank with wet transfer buffer at 1.0A for 90 minutes). For proteins of a lower apparent molecular weight, semi-dry transfer was used (Thermo Scientific Owl HEP series electroblotting system with semi-dry buffer ((48 Mm Tris base, 39 mM Glycine, 0.0375% SDS, 20% methanol) at 500 mA for 30 minutes). After transfer, the membrane was incubated for 5 minutes with Ponceau S solution (1% Ponceau S tetrasodium (w/v), 5% acetate(v/v)) to test the transfer. The membrane was then blocked with 5% milk powder in TBS-T for 45-60 minutes. Then, the membrane was incubated for 1 hour in primary antibody (the list of primary and secondary antibodies can be found in Supplementary Table 2 as *Extended data* ( Zegerman, 2023))., washed 3 times with TBS-T, incubated for 1 hour in secondary antibody, washed 2 times with TBS-T and then 1 time with TBS. All washes were done for 5–10 minutes. The membrane was then incubated for 1 minute in Amersham ECL Western Blotting Detection Reagent or for 5 minutes in Amersham ECL prime Western Blotting Detection Reagent. The membrane was then held in a film cassette with an Amersham Hyperfilm for 1-15 minutes depending on the protein. The film was visualised using a blot imaging machine.

### TurboID preparation of whole cell lysates from yeast

A total of 25-50 ml cell cultures of density 2x10 ^7^ cells/ml were pelleted and resuspended in 950 μl Zymolyase digestion buffer (50 mM Tris pH 7.5, 1 M sorbitol, 10 mM β-mercaptoethanol, protease inhibitors). Then, 100 μl of Zymolyase 20T solution was then added (10 mg/ml of Zymolyase 20T in Zymolyase digestion buffer) and the solution gently shaken at 37°C for 5 minutes. After testing microscopically for the creation of spheroplasts, the mixture was spun down at 1,500 g (4,000 rpm on desktop centrifuge). The supernatant was removed and the pellet was resuspended in 400 μl of SDS lysis buffer (3% SDS, 50 mM Tris pH 8.8, 10 mM DTT) After testing microscopically for lysis, the solution was boiled at 99°C for 10 minutes, cooled down at room temperature and made up to 2 ml with modified RIPA buffer (50 mM Tris pH 7.5, 150 mM NaCl, 0.5% Sodium deoxycholate, 1% Triton X-100). The cell lysate was pre-cleared by spinning the solution at 13.3 krpm for 15 minutes. The supernatant was then loaded on pre-washed Zeba™ Spin Desalting Columns, 7K MWCO, 5 mL to remove any excess biotin and the protein concentration was normalised between samples using A280 nm absorbance. After some of the sample was kept as input control, 50 μl of pre-washed streptavidin magnetic beads (Dynabeads *MyOne Streptavidin* C1) were added to 10 mg of protein lysate and put on a rotator for 3 hours at room temperature. The beads were washed sequentially: with RIPA (0.5% SDS, 50 mM Tris pH 7.5, 150 mM NaCl, 0.5% Sodium deoxycholate, 1% Triton X-100), 1 M KCl, 0.1 M Sodium bicarbonate, urea buffer (2 M Urea, 10 mM Tris pH 8), RIPA and thrice with 50 mM Ammonium bicarbonate. The samples were then split for mass spectrometry and western blot processing. For western blot processing, the beads were incubated with 50 μl 1x BXT elution buffer (IBA-lifesciences) for 10 minutes. A total of 16.6 μl of 4x Laemmli loading buffer (250 mM Tris pH6.8, 2% SDS, 40%(v/v) glycerol, 1%(w/v) Bromophenol blue, 20% β-mercaptoethanol) was then added and the beads were boiled at 99°C for 10 minutes. The beads were then removed. For mass spectrometry analysis, the beads were resuspended in 100 μl of 50 mM ammonium bicarbonate and flash frozen in dry ice.

### TurboID preparation of chromatin lysates from yeast

In total, 25–50 ml of cell culture with density at 1-2×10 ^7^ cells/ml, was pelleted and resuspended in 12.5 ml of solution 1 (0.1 M PIPES/KOH pH 9.4, 10 mM DTT) and incubated at 30°C for 10 minutes. The cells were pelleted and resuspended in 5 ml of solution 2 (2xSC media with 4% glucose, 0.6 M Sorbitol, 25 mM Tris, pH 7.5). Then, 25 μl of lyticase (1.2 M sorbitol, 0.1 M, Sodium Phosphate pH 7.4, 50% (v/v) glycerol, 50 μl/ml β-mercaptoethanol, 40 KU/ml Lyticase (L5263, Sigma) was added and the solution was incubated at 30°C for 5 minutes with mild agitation. Spheroplast formation was checked microscopically and the cells were then pelleted at 200 g (~1,000 rpm on bench centrifuge) for 3 minutes. The pellet was resuspended in solution 3 (2 x SC media with 4% glucose, 0.7 M Sorbitol, 25 mM Tris, pH 7.5) and incubated at 30°C for 10 minutes with mild agitation. The spheroplasts were pelleted again at 200 g lysed in 400 μl of RIPA buffer (0.5% SDS, 50 mM Tris pH 7.5, 150 Mm NaCl, 0.5% Sodium deoxycholate, 1% Triton X-100). The rest of the protocol is the same as bead enrichment using whole cell lysates.

### Mass spectrometry

Bead-bound proteins were prepared for mass spectrometric analysis by in solution enzymatic digestion. Briefly, bead-bound proteins in 40 μl of 50 mM NH4HCO3 were reduced in 10 mM DTT, and then alkylated with 55 mM iodoacetamide. After alkylation, 0.5 μg of Trypsin (Promega, UK) was added and the proteins digested for 1 h at 37°C in a thermomixer (Eppendorf, Germany), shaking at 800 rpm. Following this initial digestion, a further 1 μg of Trypsin (Promega, UK) was added and digestion continued overnight at 37°C. After digestion, 1 μl of formic acid was added and the beads centrifuged for 30 seconds at 14,000 rpm. The supernatant was then removed into a fresh, labelled tube. The resulting peptides were analysed by nano-scale capillary LC-MS/MS using an Ultimate U3000 HPLC (ThermoScientific Dionex, USA) to deliver a flow of approximately 300 nl/min. A C18 Acclaim PepMap100 5 μm, 100 μm × 20 mm nanoViper (ThermoScientific Dionex, USA), trapped the peptides before separation on a C18 BEH130 1.7 µm, 75 µm x 100 mm analytical UPLC column (Waters, UK). Peptides were eluted with a gradient of acetonitrile. The analytical column outlet was directly interfaced *via* a nano-flow electrospray ionisation source, with a quadrupole Orbitrap mass spectrometer (Q-Exactive HFX, ThermoScientific, USA). MS data were acquired in data-dependent mode using a top 10 method. High-resolution full scans (R=120 000, m/z 300–1,800) were recorded in the Orbitrap followed by higher energy collision dissociation (HCD) (26% Normalized Collision Energy) of the 10 most intense MS peaks. The fragment ion spectra were acquired at a resolution of 50,000 and dynamic exclusion window of 20 seconds was applied.

LC-MS/MS data were then searched against a protein database (UniProtKB (RRID:SCR_004426)) using the Mascot search engine programme (RRID:SCR_014322) (Matrix Science, UK) ( Perkins *et al.*, 1999). Database search parameters were set with a precursor tolerance of 10 ppm and a fragment ion mass tolerance of 0.8 Da. One missed enzyme cleavage was allowed and variable modifications for oxidized methionine, carbamidomethyl cysteine, pyroglutamic acid, phosphorylated serine, threonine and tyrosine. MS/MS data were validated using the Scaffold programme (Proteome Software Inc., USA) ( Keller *et al.*, 2002). An alternative freely available alternative to replicate our study is OpenMS (RRID:SCR_012042). All data were additionally interrogated manually.

### Mass spectrometry label-free quantification (LFQ)

LFQ intensities for each protein were collected and normalised by dividing the LFQ value over the LFQ value of the endogenously biotinylated protein Acc1. LFQ intensities equal to zero were converted to 1 so that no issues (division by 0) arose when generating the ratios (see equations below). Then, an averaging of the LFQ intensity of each protein was calculated over the number of experiments executed for each of the three different samples; no TurboID + HU (sample 1), TurboID no HU (sample 2) and TurboID + HU (sample 3). The calculations are summarised below.


$$Normalised\;protein\;X_{\left(sample\;1\right)}=\frac{protein\;X_{\left(sample\;1\right)}}{control\;protein_{\left(sample\;1\right)}}$$

$$Sample\;1,2\;or\;3\;average\;protein\;X_{\left(n\;experiments\right)}=\frac{sum(normalised\;protein\;X_{\left(experiment\;1\;to\;n\right)})}{n}$$

$$Enrichment\;(\mathrm{TurboID}\;\mathrm{vs}\;\mathrm{no}\;\mathrm{Turbo}\;\mathrm{ID})=\frac{sample\;3\;average\;protein\;X}{sample\;1\;average\;protein\;X}\;\;\;\;\;\;\;\;\;\;\;\;y\text{-}axis$$

$$Enrichment\;(\mathrm{HU}\;\mathrm{vs}\;\mathrm{no}\;\mathrm{HU})=\frac{sample\;3\;average\;protein\;X}{sample\;2\;average\;protein\;X}\;\;\;\;\;\;\;\;\;\;\;\;x\text{-}axis$$

X refers to any protein found during the mass spectrometry runs. Control protein refers to the FQ value of Acc1. Experiment 1 to n refers to the experimental repeats conducted. Enrichment is the log value of the respective ratios.

### Bimolecular fluorescence complementation (BiFC)

During timecourse experiments, 500 μl of cell culture of density 2x10 ^7^ cells/ml was pelleted and resuspended in 500 μl of PBS (+MMS/HU depending on the experiment). The solution was sonicated for 8 seconds at 40% intensity and 350 μl was loaded onto concanavalin A-coated Glass Bottom Microwell dishes (MatTek) for 5 minutes to allow cell attachment. The solution was then aspirated, and the plate covered in 2 ml PBS (+MMS/HU). Images were captured using either a Zeiss 880 Airyscan inverted confocal or a Leica SP5 confocal microscope and analysed using Fiji 2.13.1 (RRID:SCR_002285). Nuclear fluorescence was calculated manually or using a custom plug-in in Fiji. For manual calculations, Corrected Total Nuclear Fluorescence, a measurement of nuclear fluorescence correcting for any background, was used. To calculate the Corrected Total Nuclear fluorescence, nuclei (visualised by HTB2-mCherry) and cytoplasmic background were manually circled in ImageJ (equation used: Corrected Total Nuclear fluorescence = Integrated Density – (Area of selected cell * Mean fluorescence of nuclear background), adapted from https://theolb.readthedocs.io/en/latest/imaging/measuring-cell-fluorescence-using-imagej.html). For automatic calculation of nuclear fluorescence, a custom Fiji script was used. The script was generated by Richard Butler (Gurdon Institute Imaging Facility).

### Yeast two hybrid analysis

Standard yeast transformation of L40 diploid strain was executed with equimolar amounts of “bait” and “prey” plasmids. Yeast was grown on non-selective (-L -T) plates. Colonies were then spread on non-selective (-L-T) and selective (-L-T-H) plates and left to grow for 3 days.

### Peptide pulldowns

Protein Lysate preparation was done as before ( Can *et al.*, 2019). GST or FHA1-GST were expressed overnight in BL21 bacteria in 2TY and 0.5 mM IPTG. A total of 25 ml culture was spun down and then frozen. The pellet was sonicated in extraction buffer (20 mM Tris pH 8.0, 5% glycerol, 300 mM NaCl, 10 μg/ml leupeptin, 10 μg/ml pepstatin, 10 mM Benzamidine-HCl, 1 mM PMSF, PhosStop (Roche, 1 tablet / 100 ml). After centrifugation, supernatants were used in pulldowns as described below.

In total, 40 μl **Dynabeads** MyOne Streptavidin **C1** beads were washed and then incubated with 40 μl of peptide (1 mg/ml, lyophilised powder dissolved in ultrapure H _2_O). Beads were then washed and blocked with BSA (5 mg/ml)-HBS buffer (10 mM HEPES, pH 7.4, 3 mM EDTA, 300 mM NaCl) for 30 minutes (3 times, 10 minutes each). Then, beads were added to 500 μl of 2 x HBS buffer and 500 μl of protein lysate. They were incubated at room temp for 30 minutes. The beads were then washed 3 times with HBS for 5 minutes each time and then 2 times with 450 mM NaCl for 10 minutes each time. The beads were then boiled for 10 minutes with 90 μl HBS and 30 μl of 4 x Laemmli. The SDS-PAGE was run and the gel was then incubated in Bradford dye (Quick Start™ Bradford Dye Reagent 1x, Bio-Rad) for 15 minutes at room temperature.

### Transferase-Activated End Ligation sequencing (TrAEL-seq) library construction and processing

A total of 1 × 10 ^7^ cells fixed in ethanol were embedded in agarose plugs and processed into TrAEL-seq libraries as previously described ( Kara *et al.*, 2021), and libraries were sequenced on an Illumina NextSeq 500 as high-output 75-bp single end by the Babraham Institute Next-Generation Sequencing facility, before read processing as described ( Kara *et al.*, 2021).

Sequencing data are deposited at GEO, accession GSE235881 ( Hadjicharalambous *et al.*, 2023).

De-duplicated mapped reads were imported into SeqMonk (RRID:SCR_001913) v1.48 and immediately truncated to 1 nucleotide at the 5′ end, representing the last nucleotide 5′ of the strand break. Reads overlapping with non-single copy regions of the genome were filtered (rDNA, 2 μ, mtDNA, sub-telomeric regions, Ty elements and LTRs) and reads were then summed in running windows as described in figure legends. Total read counts across all included windows were normalized to reads per million mapped.

To determine ‘active ARS sites’, 10 Kb windows centred on ARS sites ( Liachko *et al.*, 2010) were defined and windows with the bottom 40% values were filtered. Centred on these ‘active ARS sites,’ 20 Kb windows upstream and 20 Kb downstream were defined and these probes were aligned and ordered based on the ‘Wild type – HU Block’ condition. Aligned probes with >1 peaks indicating probes containing more than one origin were removed. Loess smoothing was applied (15 neighbours with 2nd order smoothing polynomial) and the peak of TrAEL-seq signals in each probe was used to calculate the average distance of replication forks from their respective origin. Statistical analysis of the median for each biological replicate was performed using ordinary one-way ANOVA with multiple comparison corrections (15 comparisons).

## Results

### Internally TurboID-tagged Rad53 is viable and active *in vivo*

To discover new mechanisms of checkpoint-dependent control of DNA replication, we set out to establish a proximity labelling method to identify transient interactors of the checkpoint kinase Rad53 in budding yeast. For this, we tagged Rad53 with TurboID, which is a promiscuous form of the *E.coli* biotin ligase BirA, allowing biotin-labelling of proximal nucleophilic residues such as lysines ( Qin *et al.*, 2021). In addition, we also tagged Rad53 with Apex2, the ascorbate peroxidase enzyme that converts biotin-phenol to biotin-phenoxyl radicals in the presence of hydrogen peroxide, resulting in biotinylation of proximal electron-rich amino acid side chains, such as tyrosines ( Qin *et al.*, 2021). We and others have previously shown that tagging Rad53 at the N- or the C-terminus generates hypomorphic mutants of Rad53 and reduces the levels of this protein ( Can *et al.*, 2019; Conde *et al.*, 2010). Therefore, we decided to identify internal positions in Rad53 that could be used for inserting these biotinylation enzymes, without disrupting protein function.

**Figure 1.**
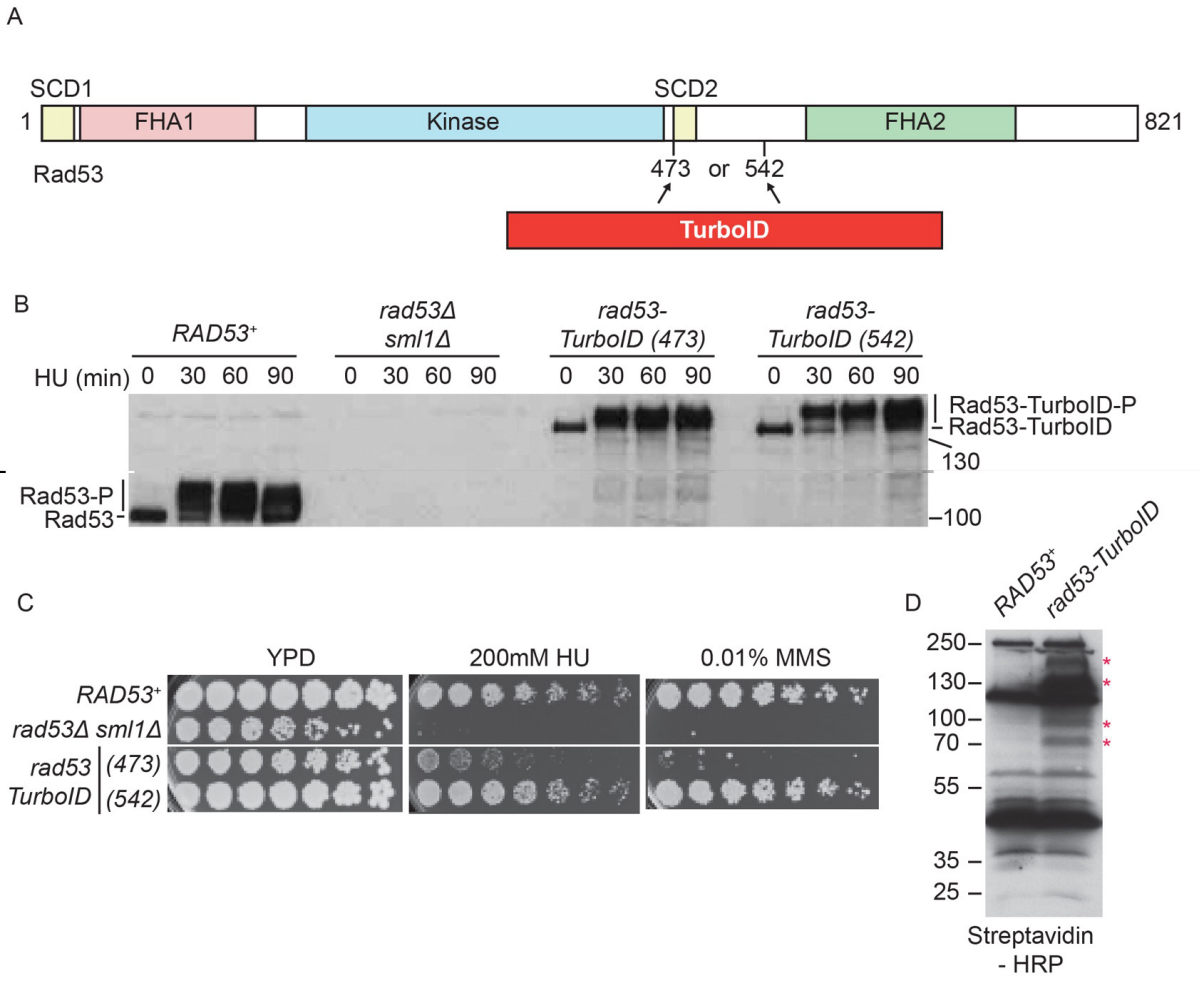
Internally tagged Rad53-TurboID is functional *in vivo*. **A**) Scale diagram of the Rad53 checkpoint kinase from budding yeast. SCD = (S/T)Q cluster domain, FHA = Forkhead-associated domain. TurboID was inserted into endogenous Rad53 at either amino acid 473 or 542. **B**) Western blot of Rad53 from the indicated strains, arrested in G1 phase with alpha factor (0) and released into S-phase with 200 mM HU for the indicated time. The internally tagged *rad53-TurboID* alleles, replace the *RAD53* gene, at the endogenous locus. **C**) Growth assays of the indicated yeast strains on YPD media, with or without genotoxic agents. Image taken at 48 hours. **D**) Anti-biotin western blot of yeast extracts from the indicated strains, using streptavidin-HRP as a probe. From here on, *rad53-TurboID* refers to *rad53-TurboID (542)*. * Marks bands that appear specifically in the *rad53-TurboID* strain. Bands that appear in both strains, are likely to be endogenously biotinylated proteins.

Rad53 contains two phospho-threonine-binding forkhead-associated (FHA) domains, FHA1 and FHA2, a central serine/threonine kinase domain and two (S/T)Q cluster domains (SCD, Figure 1A), which are phosphorylated by Mec1/Tel1 *in vivo* ( Chen *et al.*, 2014). To avoid causing structural changes or interfering with the phosphorylation of Rad53, we inserted the proximity labelling enzyme either before (amino acid 473) or after the SCD2 (amino acid 542). To address whether the internally tagged Rad53-TurboID fusions were functional *in vivo*, we first analysed the phosphorylation shift of the protein, indicating Rad53 activation, after replication stress ( Figure 1B). We induced replication stress in synchronised cultures by inhibiting ribonucleotide reductase with hydroxyurea (HU), causing the depletion of dNTPs. In *RAD53* wild type cells, Rad53 protein becomes phosphorylated and activated as cells enter S-phase in the presence of HU, as expected ( Figure 1B). Both internally tagged versions of Rad53-TurboID were also phosphorylated in HU, although Rad53-TurboID inserted at amino acid 542, showed slightly greater activation than the insertion at 473 ( Figure 1B). Since Rad53 is essential for cells to survive DNA damage and replication stress, we also tested whether these internally tagged *RAD53* alleles affected cell growth. While the *rad53-TurboID (542)* allele did not affect cell growth in the presence of HU or the DNA alkylating agent methyl methanesulfonate (MMS, Figure 1C), *rad53-TurboID (473)* showed significant lethality in the presence of these genotoxins, similar to strains completely lacking Rad53 ( *rad53∆*). This growth assay strongly suggests that *rad53-TurboID (473)* is a hypomorph, while *rad53-TurboID (542)* is functional. Rad53 containing Apex2 at either amino acid 473 or 542 also appeared to be functional *in vivo* (Supplementary Figure 1 in *Extended data* ( Zegerman, 2023)).

While TurboID can utilise endogenous biotin for proximity labelling ( Qin *et al.*, 2021), Apex2 requires the addition of biotin-phenol as a substrate, which does not readily penetrate the yeast cell wall ( Hwang & Espenshade, 2016). As a result, we could detect specific biotinylated bands in whole extracts of cells containing Rad53-TurboID (542) ( Figure 1D), but we could not detect biotinylated proteins after addition of biotin-phenol to partially permeabilised yeast cells containing Rad53-Apex2 (blank gel, data not shown). Therefore, we decided to analyse Rad53 interactors using Rad53-TurboID (542), hereafter called Rad53-TurboID, as this fusion showed both normal physiological activity ( Figure 1B and C) and protein biotinylation *in vivo* ( Figure 1D).

### Identification of novel Rad53-proximal proteins using TurboID

**Figure 2.**
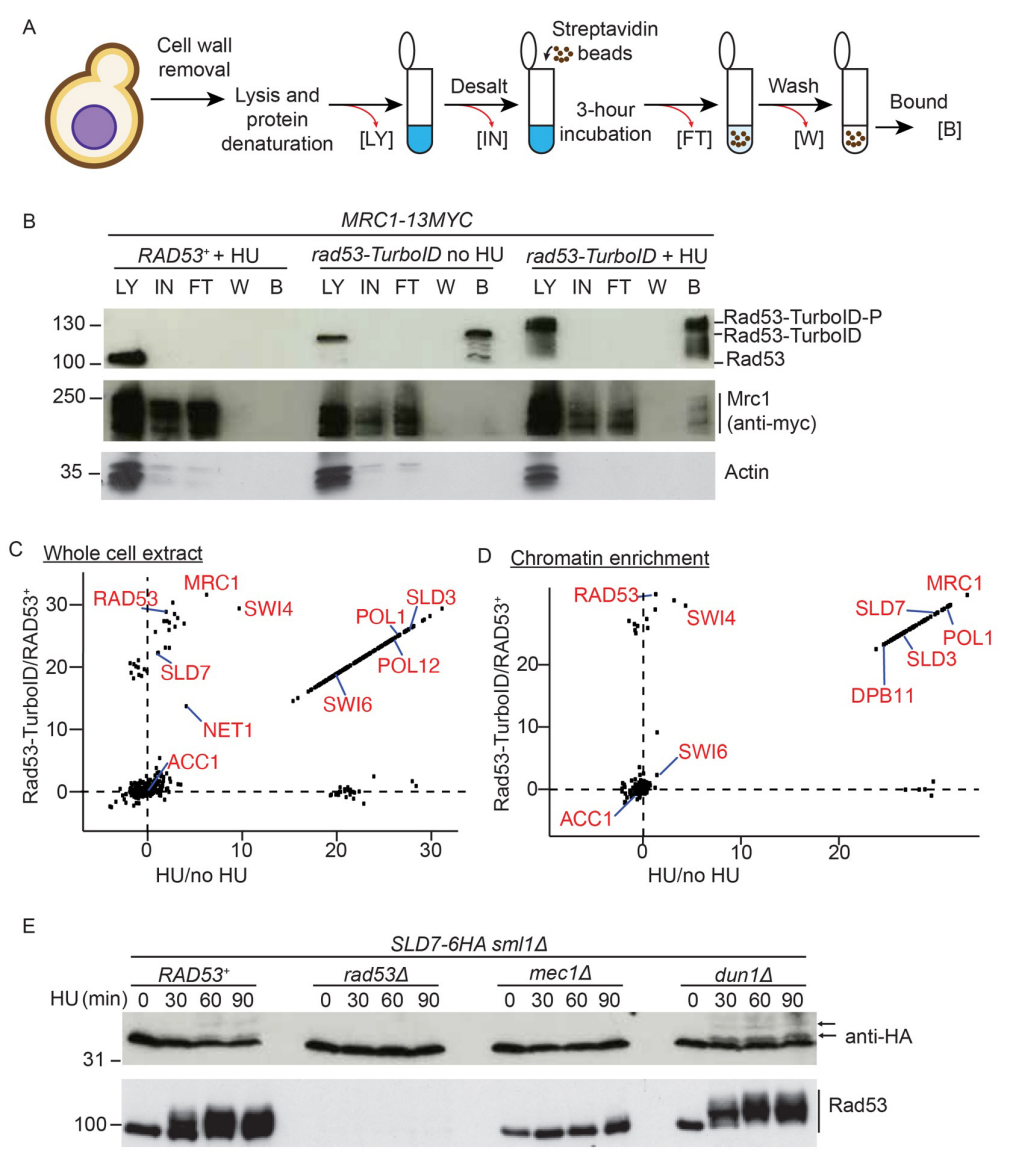
*In vivo* biotinylation strategy identifies new Rad53-interactors. **A**) Protocol for isolation of biotinylated proteins from yeast. LY = lysate, IN = input, FT = flow through, W = wash, B = bound. **B**) Western blot of the biotinylated purified proteins from the indicated strains, released from G1 phase into either a normal S-phase (no HU) or S-phase in the presence of 200 mM HU (+ HU). **C**) Label-free quantification (LFQ) analysis of mass spectrometry results from the whole cell extracts. The analysis compares HU treated *versus* untreated on the x-axis and the Rad53-turboID tagged strain *versus* the untagged wild type strain on the y-axis. Each dot represents a different statistically significant protein hit. Some key replication factors and known Rad53 interactors/substrates are indicated in red. Acc1, acetyl-CoA carboxylase, is a biotin containing enzyme and an expected hit in all conditions. LFQ values of zero were converted to 1 to allow the calculation of the ratios, which is why many of the hits lie on a diagonal line (see methods). **D**) As c), except from the chromatin enriched proteome. **E**) Western blot of HA-tagged Sld7 from the indicated strains. Lower mobility, likely phosphorylated forms of Sld7 are highlighted with arrows.

Stalling of replication forks, for example after depletion of dNTPs with HU, results in activation and recruitment of Rad53 to the replisome ( Can *et al.*, 2019). Although Rad53-TurboID can biotinylate proteins *in vivo* ( Figure 1D), we wondered whether this fusion could label Rad53 interactors at stalled forks. Using the biotinylated protein purification method delineated in Figure 2A, we first assessed whether Rad53-TurboID specifically targeted Mrc1 (Claspin), a known Rad53-interactor at stalled replisomes ( McClure & Diffley, 2021). Purification of biotinylated proteins from cells lacking Rad53-TurboID, showed that this assay is specific as we did not detect Rad53, Mrc1 or a negative control (actin) in the bound fraction ( Figure 2B, left). In Rad53-TurboID containing S-phase cells, without HU, we did detect Rad53 bound to beads, suggesting that Rad53-TurboID can biotinylate itself, but we did not detect Mrc1 or actin ( Figure 2B, middle). Importantly, in HU we not only detected the phospho-shifted Rad53-TurboID, but we also detected Mrc1 in the streptavidin-bound fraction ( Figure 2B, right), showing that Rad53-TurboID can label specific Rad53-interactors in HU. To further confirm that the biotin labelling of Mrc1 by Rad53-TurboID is specific to Rad53, not simply due to TurboID being expressed and localised to the nucleus, we performed an additional control comparing Rad53-TurboID to a nuclear NLS-GFP-TurboID. Significantly, only in the strain containing Rad53-TurboID did we detect Mrc1 bound to streptavidin beads (Supplementary Figure 2A in *Extended data* ( Zegerman, 2023)), again showing that this assay can specifically identify Rad53-interactors.

To identify Rad53-interactors in an unbiased way we performed mass-spectrometry on streptavidin purified biotinylated proteins. For this analysis we used the same approach as in Figure 2B, whereby we compared a strain with wild type *RAD53* versus *rad53-TurboID*, with and without HU. Data from this mass-spectrometry analysis (Supplementary Table 1 in *Extended data* ( Zegerman, 2023)) are presented as a comparison between Rad53-TurboID *versus* untagged Rad53 (y-axis, Figure 2C) and a comparison between HU arrested *versus* normal S-phase cells (x-axis, Figure 2C). Hits in the lower left include TurboID-independent biotinylated proteins, such as Acc1 (Acetyl-CoA carboxylase), while hits in the top left are specific to Rad53-TurboID, but are not preferentially biotinylated in HU, such as Rad53 itself ( Figure 2C). Importantly, hits above and to the right of the dotted lines are proteins that are preferentially biotinylated by Rad53-TurboID in HU ( Figure 2C). Hits in this category include several known Rad53 interactors such as the replication protein Mrc1, the transcription factor Swi6 and the nucleolar protein Net1 ( Smolka *et al.*, 2006). To further increase the specificity of this approach for identifying new replication targets of Rad53, we performed the same experiment as in Figure 2A, but after cell lysis we separated the chromatin fraction from the cytosolic fraction using a detergent-based method ( Desdouets *et al.*, 1998). This method indeed enriched Rad53-replisome interactors in HU including Mrc1 ( Figure 2D; Supplementary Table 1 in *Extended data* ( Zegerman, 2023)).

TurboID causes labelling of direct interactors but the biotinylation radius of TurboID can also lead to labelling of proteins that are proximal to true interactors ( Qin *et al.*, 2021). For example, Swi6, which is a known interactor with Rad53 ( Sidorova & Breeden, 1997; Smolka *et al.*, 2006), binds to the transcription factor Swi4 and both proteins were identified as hits in this TurboID analysis ( Figure 2C–D). As the streptavidin pulldowns are performed on denatured protein samples ( Figure 2A), Swi4 is not enriched by virtue of binding to Swi6, but instead is likely to be biotinylated by Rad53-TurboID.

Swi6 not only binds to Rad53 but is also a Rad53 substrate ( Sidorova & Breeden, 1997; Smolka *et al.*, 2006). In addition, Sld3 is a substrate of Rad53 ( Lopez-Mosqueda *et al.*, 2010; Zegerman & Diffley, 2010) and is a prominent TurboID hit ( Figure 2C–D), but does not directly interact with Rad53 ( Can *et al.*, 2019). We therefore wondered whether, in similarity to Sld3 and Swi6, Rad53-TurboID hits might also be Rad53 substrates. The Sld3-binding protein Sld7 is enriched as a Rad53-proximal protein ( Figure 2C–D) and the human orthologue of this protein, MTBP, has recently been shown to be phosphorylated in a checkpoint kinase-dependent manner in human cells ( Ferreira *et al.*, 2021). Analysis of Sld7 phosphorylation using phos-tag gel electrophoresis, demonstrated that in wild type cells at least two lower mobility forms of Sld7 can be detected in the presence of HU ( Figure 2E), which were not present in cells lacking Rad53 activation ( *mec1∆*) or in *RAD53* null cells, but were apparent in cells lacking the Dun1 kinase, which is downstream of Rad53 ( Figure 2E). This experiment suggests that the Sld3-binding protein Sld7 is also likely to be a substrate of Rad53 *in vivo*.

Comparison of the Rad53-TurboID hits from this study with existing phospho-proteomic studies with Rad53 (citations for these studies are as follows: Bastos de Oliveira *et al.*, 2015; Chen *et al.*, 2010; Ho *et al.*, 2002; Hustedt *et al.*, 2015; Lao *et al.*, 2018; Smolka *et al.*, 2007; Smolka *et al.*, 2006; Zhou *et al.*, 2016), showed only a small degree of overlap between these approaches (Supplementary Figure 2B in *Extended data* ( Zegerman, 2023)), suggesting that Rad53-TurboID hits are not necessarily Rad53 substrates. Together, these experiments demonstrate that TurboID can identify interactors and substrates of Rad53 *in vivo*.

### DNA polymerase alpha interacts with Rad53 *in vivo*

**Figure 3.**
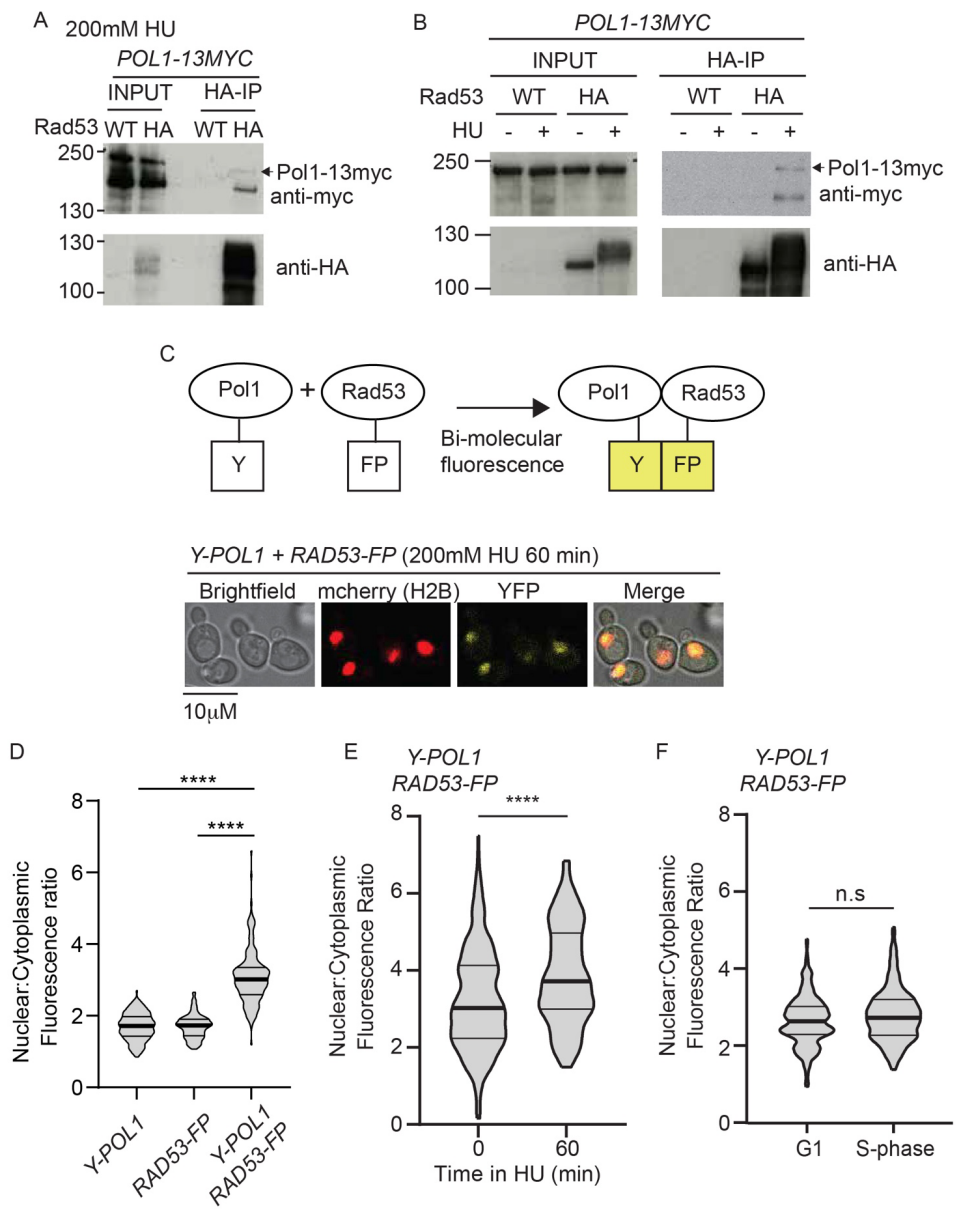
Rad53 interacts with Polymerase alpha *in vivo*. **A**) Anti-HA immunoprecipitation (IP) from a *RAD53* wild type (WT) strain or a strain containing internally tagged Rad53-6HA (HA). All strains contain *POL1-13myc* and were arrested in 200 mM HU. **B**) As a), except IP performed from strains released from G1 phase into S-phase in the presence (+) or absence (-) of HU. **C**) Top, schematic diagram of bi-molecular fluorescence assay. Endogenous Pol1 was N-terminally tagged with a fragment of YFP (Y), while Rad53 was internally tagged with the remaining fragment of YFP (FP). Interaction between Pol1 and Rad53 would bring together the two halves of YFP to allow fluorescence (in yellow). Bottom, example images of yeast containing these YFP fragment tagged Pol1/Rad53 constructs treated with 200 mM HU for 60 minutes after release from G1 phase. Strains also contain H2B-mcherry to delineate the nucleus. **D**) Violin plot of the nuclear to cytoplasmic fluorescence ratio of strains containing the individual YFP fragment alleles or both. These cells were released from G1 phase into 200 mM HU for 60 minutes. n>150, p-value from a t-test **** < 0.0001. **E**) Violin plot of the YFP fluorescence ratio, as in d) from cells containing both Y-Pol1 and Rad53-FP, released from G1 phase (0) into 200 mM HU for 60 minutes. n>100, p<0.0001 (****). **F**) As e), except cells were released into S-phase in the absence of HU for 60 minutes. n>150, n.s = not significant.

The DNA polymerase subunit of Pol α (Pol1), the enzyme responsible for priming leading and lagging strand synthesis, was identified by TurboID as a potentially novel Rad53 interactor in both whole cell extracts and on chromatin ( Figure 2C–D). To confirm that Pol1 interacts with Rad53 we performed co-immunoprecipitations, which demonstrated that Rad53 interacts with Pol1 in HU ( Figure 3A), but less so in the absence of HU ( Figure 3B). To assess this interaction by another method, we used bi-molecular fluorescence complementation (BiFC, Sung & Huh, 2007), whereby Pol1 and Rad53 were separately tagged with parts of YFP, which only fluoresce when the two parts of YFP are brought together ( Figure 3C). N-terminal tagging of Pol1 and internal tagging Rad53 (as for TurboID, Figure 1) with these split fragments of YFP did not affect the essential functions of these proteins (Supplementary Figure 3 in *Extended data* ( Zegerman, 2023)). Significantly, YFP fluorescence was only observed in yeast that contain both Pol1 and Rad53 tagged with the split fragments of YFP ( Figure 3C–D), suggesting that these two proteins indeed interact *in vivo*. As was the case for the Co-IPs ( Figure 3A–B), the BiFC between Pol1 and Rad53 was enhanced in cells arrested in HU rather than a normal S-phase ( Figure 3E–F). These interaction analyses support the TurboID data that Pol1 and Rad53 interact, preferentially in cells with stalled replisomes.

### DNA polymerase alpha is phosphorylated by CDK not checkpoint kinases *in vivo*

**Figure 4.**
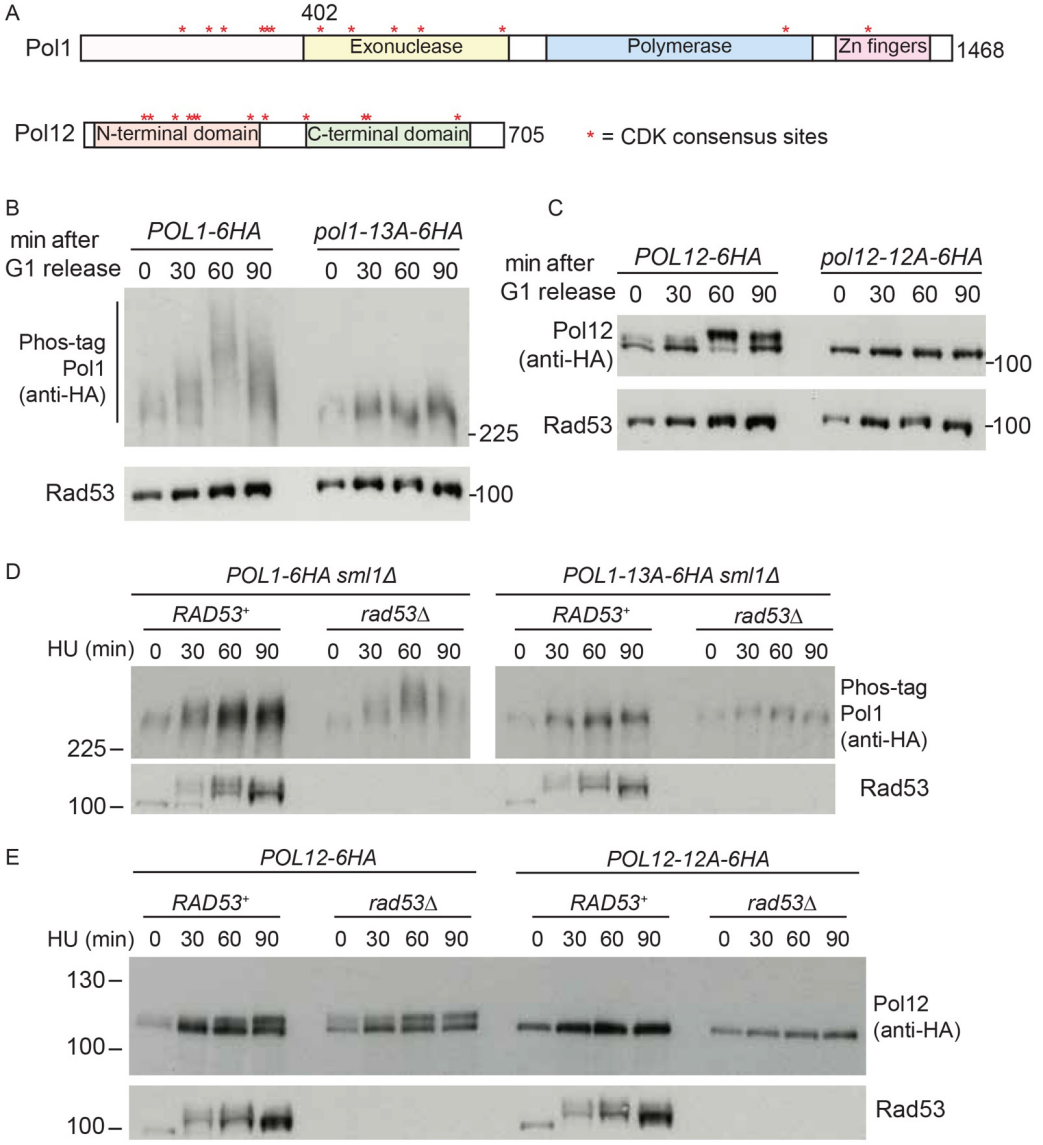
Polymerase alpha subunits Pol1 and Pol12 are CDK phosphorylated in S-phase. **A**) Scale diagram of the two largest subunits of polymerase alpha from yeast. * refers to CDK consensus sites. **B**) Western blot of the indicated strains released from G1 phase (0) into S-phase for the indicated times. All 13 serines/threonines within CDK consensus sites are mutated to alanine in the *pol1-13A* mutant. **C**) As b), except with the Pol12 tagged strains. All 12 serines/threonines within CDK consensus sites are mutated to alanine in the *pol12-12A* mutant. **D**) As b), except cells were released from G1 phase into S-phase with 200 mM HU. **E**) As c), except cells were released from G1 phase into S-phase with 200 mM HU.

As TurboID identified Rad53 interactors, such as Pol1 ( Figure 3), and also substrates ( *e.g.*, Sld7, Figure 2E), we set out to determine whether Rad53 not only binds to, but also phosphorylates DNA polymerase alpha. Both Pol1 and the non-catalytic B-subunit of Pol α, Pol12, have been shown to be phosphorylated by CDK *in vivo* ( Desdouets *et al.*, 1998; Holt *et al.*, 2009; Pellicioli *et al.*, 1999). Pol1 and Pol12 have 13 and 12 potential CDK sites (SP/TP), respectively, ( Figure 4A) and to demonstrate their role in Pol α phosphorylation we generated alleles of both Pol1 and Pol12 that contain alanine mutations in the serines/threonines of all their CDK sites ( *pol1-13A* and *pol12-12A*). Notably yeast containing these alleles are viable ( *e.g.*, Figure 4B–C), showing that these CDK sites are not required for any essential functions of DNA polymerase alpha.

While wild type Pol1 and Pol12 become phosphorylated upon entry into S-phase, consistent with the timing of activation of CDK, Pol1-13A and Pol12-12A were not detectibly phosphorylated under these conditions ( Figure 4B–C). To determine whether Pol1 and Pol12 are phosphorylated by Rad53, we performed the same experiment but in cells released from G1 phase into HU to stall forks globally and activate Rad53 ( Figure 4D–E). In HU, neither Pol1-13A nor Pol12-12A were visibly phosphorylated, either in the presence or absence of Rad53 ( Figure 4D–E), suggesting that only the CDK sites contribute to Pol1/Pol12 phosphorylation in HU. We obtained a similar result when we analysed wild type Pol1/Pol12 in HU arrested cells in which CDK is inhibited (Supplementary Figure 4A-B in *Extended data* ( Zegerman, 2023)).

Consistently with previous reports ( Palou *et al.*, 2015; Pellicioli *et al.*, 1999), Pol1 and Pol12 actually appeared to be more phosphorylated in cells that lack Rad53 ( Figure 4D–E) or the upstream kinase Mec1 (Supplementary Figure 4C-D in *Extended data* ( Zegerman, 2023)). One possible explanation for this is that Mec1/Rad53 may inhibit CDK activity by multiple mechanisms ( Palou *et al.*, 2015), leading to higher CDK activity and therefore more Pol1/Pol12 phosphorylation in *mec1∆* or *rad53∆* cells. Together these data confirm that both Pol1 and Pol12 are CDK substrates *in vivo*, but it is not likely that these proteins are phosphorylated by the checkpoint kinases.

### Rad53 interacts with a CDK phosphorylated site in Pol1

**Figure 5.**
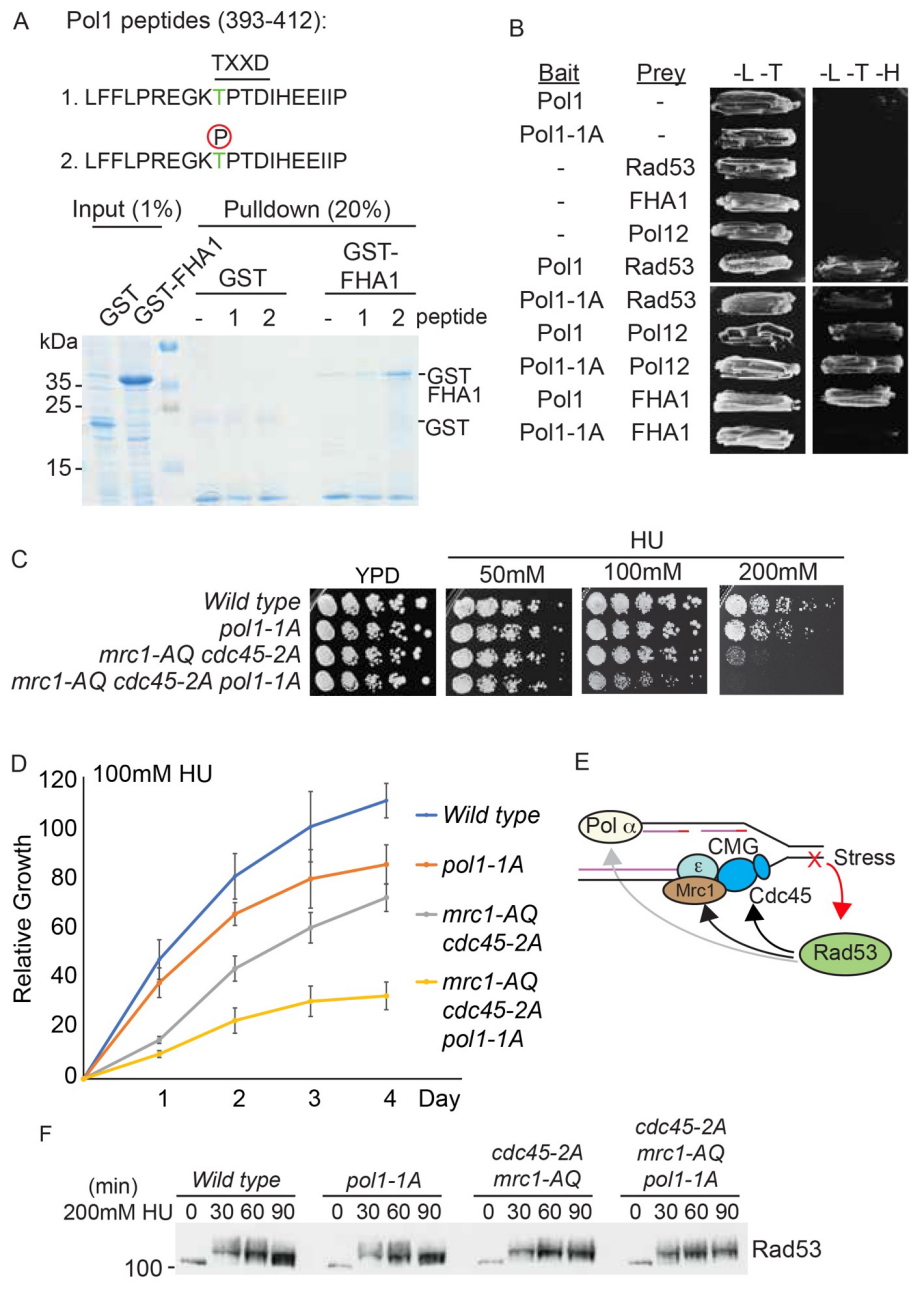
Interaction between phosphorylated residue T402 in Pol1 and Rad53 FHA1 is required for viability in HU. **A**) Top, sequence of the Pol1 peptides (393-412) containing the putative FHA binding consensus TXXD, which also contains a CDK consensus site (TP). Peptide 1 is unphosphorylated, peptide 2 contains phospho-threonine at position 402. These peptides contain a N-terminal biotin moiety (not shown). Bottom, Coomassie stain of a streptavidin pulldown of the biotinylated Pol1 peptides incubated with *E. coli* whole cell extracts expressing either GST or GST fused to the Rad53 FHA1 domain (1-165). **B**) Yeast two-hybrid analysis of the indicated bait and prey proteins (- indicates empty vector control). All proteins are full length, except for the FHA1 domain of Rad53 (1-165). Pol1-1A contains a T402A mutation at the TXXD consensus site. -L-T media is non-selective, while -L-T-H media is selective for interacting proteins. **C**) Growth assays of the indicated yeast strains on YPD media, with or without the addition of HU. All plates were grown at 30°C and this image was taken at 48 hours. The *mrc1-AQ* mutant has all 17 Mec1/Tel consensus phosphorylation sites mutated to alanine and the *cdc45-2A* mutant has the Rad53 interaction sites (T189/T195) mutated to alanine. **D**) Quantitation of the 100 mM HU plate growth assay in c) over time. Error bars are SD, n=3. **E**) Schematic diagram showing interactions of Rad53 with components of the leading strand machinery (Cdc45/Mrc1) and the lagging strand machinery (Pol alpha). Rad53 binds to Cdc45 and Mrc1 and also phosphorylates them (black arrows), while Rad53 binds to, but does not phosphorylate Pol1 (grey arrow). **F**) Rad53 western blot of the indicated strains released from G1 phase into S-phase with 200mM HU.

Rad53 interacts with phosphorylated proteins, such as Cdc45 and Mrc1, *via* its FHA domains ( Can *et al.*, 2019; Smolka *et al.*, 2006). Since Pol1 is phosphorylated by CDK we wondered whether these phosphorylations might be important for the interaction with Rad53. The Rad53 FHA1 domain preferentially interacts with phospho-threonines within pTXXD motifs, while FHA2 preferentially binds to pTXXI motifs ( Durocher *et al.*, 2000). Significantly, while there are no TPXI motifs in Pol1, the CDK site at amino acid 402 (TPXD) forms a potential FHA1 interaction motif ( Figure 5A). To determine whether this site can interact with the FHA1 domain of Rad53, we performed GST pulldowns with Pol1 peptides and the Rad53 FHA1 domain expressed in bacteria. While the unphosphorylated Pol1 peptide (amino acids 393-412) did not bind to the FHA1 domain, the phosphorylation of T402 resulted in specific interaction with FHA1 ( Figure 5A). To determine whether T402 mediates the interaction between Pol1 and Rad53 in yeast, we performed a yeast two-hybrid analysis. Wild type full length Pol1 interacts with both full length Rad53 and the Rad53 FHA1 domain alone ( Figure 5B). Importantly, mutation of T402 to alanine (hereafter called Pol1-1A), greatly reduced the interaction between Pol1 and full length Rad53, as well as with the FHA1 domain alone, but critically did not affect the interaction with Pol12 ( Figure 5B). These data show that the interaction between Pol1 and Rad53 is at least in part mediated by Rad53 FHA1-mediated binding to the Pol1 CDK site T402.

Although Pol12, the B-subunit of DNA polymerase α, which interacts with Pol1, was identified by TurboID in whole cell extracts ( Figure 2C), we did not detect any interaction between Pol12 and Rad53 by yeast two-hybrid analysis and Pol12 has no TPXI or TPXD motifs. Therefore, we consider it likely that Pol12 was targeted by Rad53-TurboID ( Figure 2C) due to its proximity to Pol1.

### Rad53 interaction with Pol1 is important for regulating replisome progression in response to replication stress

Having identified the site of interaction between Pol1 and Rad53 ( Figure 5A–B), we set out to determine how important this interaction is for the checkpoint response to replication stress. The Pol1-1A mutation, which abrogates the interaction with Rad53 ( Figure 5B), shows no growth defect on normal media and only a minor defect in the presence of HU ( Figure 5C–D). As there are at least two other interactions between Rad53 and the replisome, *via* Cdc45 and Mrc1 ( Figure 5E, Can *et al.*, 2019; Chen *et al.*, 2014), we wondered whether combined mutation of these different modes of interaction between Rad53 and the replisome might lead to synergistic defects in the response to replication stress. Mutation of the Mec1-phosphorylated, Rad53-interaction sites in Mrc1 ( *mrc1-AQ* mutant) and the two Rad53-interaction TXXD motifs in Cdc45 ( *cdc45-2A* mutant) together results in some sensitivity to fork stalling agents ( Figure 5C–D, Can *et al.*, 2019). Importantly, combination of the *pol1-1A* mutation with these other Rad53-interaction mutants led to significantly worse growth in the presence of HU ( Figure 5C–D). This fitness defect was not due to a defect in Rad53 activation ( Figure 5F). These data demonstrate that the interaction between Pol1 and Rad53 is important during replication stress, particularly in cells that lack other interactions between Rad53 and the replisome.

**Figure 6.**
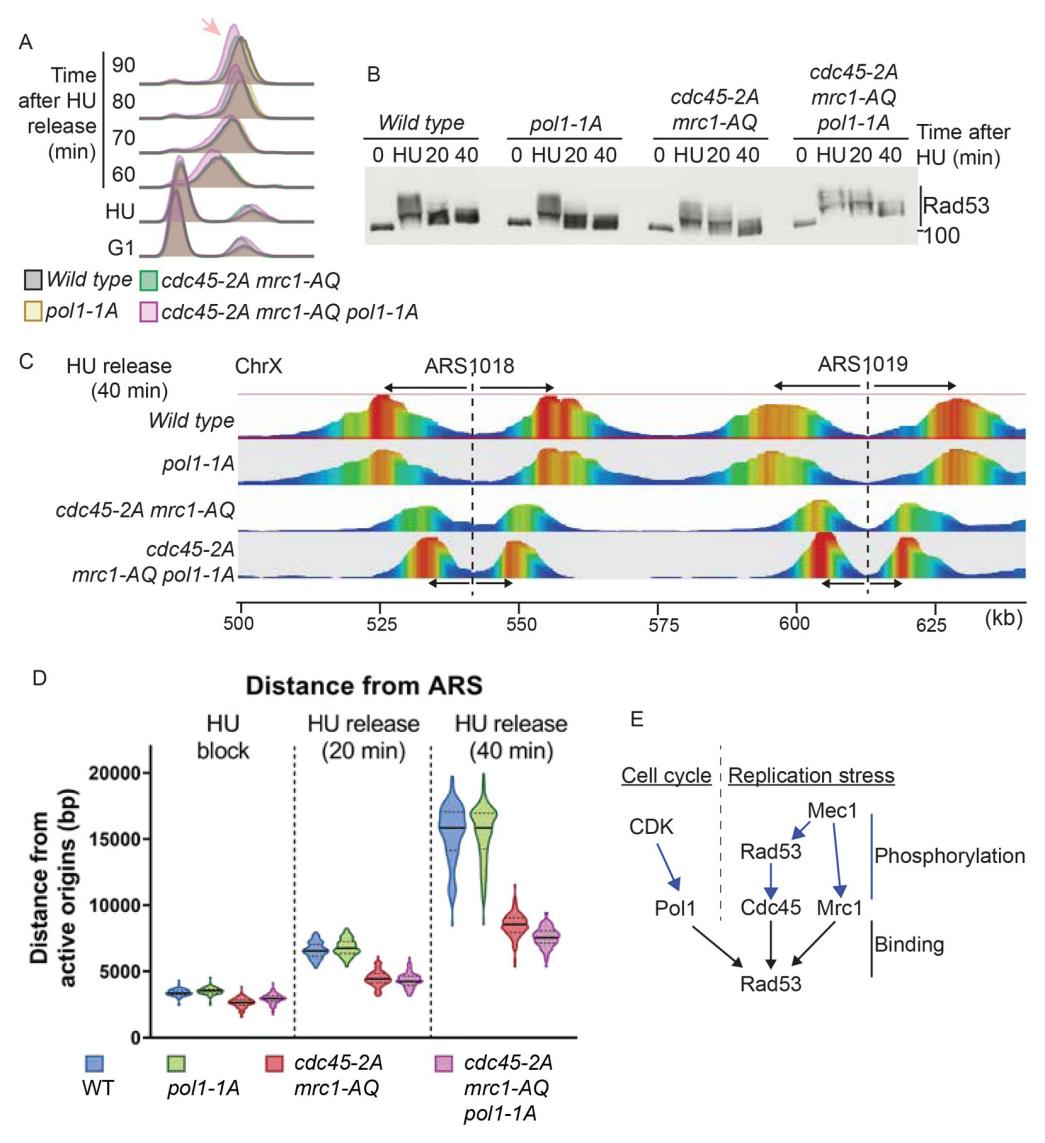
Rad53 interaction with replication proteins, including Pol1, affects fork progression. **A**) Flow cytometry of the indicated strains released from G1 phase (0) into S-phase with 200 mM HU for 30 minutes (HU), followed by washing away the HU and release. The arrow indicates a delay in S-phase completion in the *cdc45-2A mrc1-AQ pol1-1A* strain. **B**) Rad53 western blot of the experiment in a). **C**) TrAEL-seq analysis of replication forks 40 minutes after release of the indicated strains from HU arrest. Only a fragment of Chromosome X is shown here. TrAEL-seq reads are coloured by abundance from red to blue. The position of the origins ARS1018 and ARS1019 are indicated with dotted lines and the direction of movement of replication forks from the origins is indicated with arrows. **D**) Violin plot of the distance of replisomes from origins at the indicated times after release from HU. The average distance travelled by forks from each origin was calculated based on the site of maximum read count within 20 Kb of the origin. The y-axis is to the distance of each replication fork from the corresponding origin. The data here are representative from two biological repeats. The p-values from the averages of these biological replicates is in Supplementary Figure 5. **E**) Rad53 binds to at least three proteins at the replication fork; Pol1, Cdc45 and Mrc1 (black arrows). The interaction of Rad53 with Mrc1 and Cdc45 is mediated by phosphorylation of these proteins by Mec1 and Rad53 respectively (right, blue arrows). Pol1 on the other hand, is phosphorylated by CDK, which allows binding of Pol1 to Rad53 FHA1 (left). Therefore, the interactions between Rad53 and the replication machinery are regulated both by replication stress (right) and by the cell cycle (left).

Since the Pol1-Rad53 interaction contributes to cell fitness in response to HU, we wondered whether this interaction is important for replisome progression. Analysis of S-phase progression after release of cells arrested in HU, showed that the *pol1-1A* mutant strain progressed through S-phase similarly to the wild type strain, whereas the triple mutant that abrogates the Rad53 interaction with Pol1, Mrc1 and Cdc45 ( *pol1-1A mrc1-AQ cdc45-2A*) showed S-phase defects compared to the *mrc1-AQ cdc45-2A* strain ( Figure 6A). These S-phase defects were not due to defective Rad53 activation ( Figure 6B), but as in Figure 5F, we noticed that Rad53 deactivation was delayed in the *pol1-1A mrc1-AQ cdc45-2A* strain. This delay in Rad53 deactivation is consistent with continued defects in replication fork progression, even after HU has been washed away.

To assess replisome progression directly, we analysed DNA replication using TrAEL-seq, which captures the position of 3’ DNA ends across the genome at base pair resolution ( Kara *et al.*, 2021). This method preferentially labels the leading strand and provides a measurement of fork position across a population of cells. For TrAEL-seq, we synchronised cells in G1 phase, released them into 200 mM HU to arrest all forks and then we released the cells into normal media to analyse fork recovery, as in Figure 6A–B. Analysis of replication fork position after arrest and release from HU, showed that fork position and recovery in the *pol1-1A* mutant strain was very similar to wild type ( Figure 6C–D), demonstrating that Pol1 interaction with Rad53 by itself plays little role in regulating the progression of the leading strand during stress. By averaging the position of all forks across two biological repeats, we do observe a statistically significant small reduction in fork progression in the *pol1-1A* mutant strain at 40 minutes after recovery from the HU arrest (Supplementary Figure 5A in *Extended data* ( Zegerman, 2023)). This difference is unlikely to be due to a change in the number of fired origins, as we observe no difference in origin firing between the wild type and the *pol1-1A* mutant strain as determined by counting the number of peaks in the TrAEL-seq analysis (which gives a value that is twice the total number origins that have fired, as there are 2 forks per origin, (Supplementary Figure 5B in *Extended data* ( Zegerman, 2023)).

Unlike the *pol1-1A* mutant strain, the Cdc45 and Mrc1 mutants that cannot interact with Rad53, showed a major defect in fork recovery, with forks remaining closer to origins even after HU was washed away ( Figure 6C–D). A reduction in fork progression in this strain may be partially explained by the fact that the *mrc1-AQ* mutant leads to more origin firing in HU (Supplementary Figure 5B in *Extended data* ( Zegerman, 2023) and Serra-Cardona *et al.*, 2021)), but likely also reflects an important role for Cdc45 and Mrc1 in regulating leading strand progression during stress ( McClure & Diffley, 2021; Serra-Cardona *et al.*, 2021).

Importantly, in the triple mutant strain that prevents Rad53 interaction with Pol1, Cdc45 and Mrc1, we observed that forks remained slightly closer to the origins than the *cdc45-2A mrc1-AQ* double mutant alone during the period of fork recovery ( Figure 6C–D, 40 minutes time point and Supplementary Figure 5A in *Extended data* ( Zegerman, 2023)). This difference was not due to a difference in the number of fired origins comparing the *cdc45-2A mrc1-AQ* strain to the *pol1-1A cdc45-2A mrc1-AQ* strain (Supplementary Figure 5B in *Extended data* ( Zegerman, 2023)). This defect in fork recovery is consistent with the reduced total S-phase progression ( Figure 6A) and decreased cell viability in this triple mutant strain ( Figure 5C–D). Together, these data demonstrate that the Pol1-Rad53 interaction, which was identified by TurboID, indeed plays a role in regulating fork progression and cell survival in the presence of replication stress.

## Discussion

### TurboID as a method to identify new targets of the checkpoint kinases

The capacity for TurboID to capture transient and unstable interactions, including kinase-substrate interactions, provides a new method to pinpoint the functions of kinases in cells ( Niinae *et al.*, 2021). Despite the low substrate specificity and diversity of functions of Rad53 in response to replication stress and DNA damage, we have validated TurboID as an approach to identify checkpoint kinase interactors and substrates *in vivo*. We considered TurboID to be particularly suited to study Rad53 function, as Rad53 has two phospho-protein interacting FHA domains and has been shown to target substrates through specific protein interactions for example binding Cdc45 to target Sld3 ( Can *et al.*, 2019) and by directly interacting with substrates, such as Dbf4 ( Chen *et al.*, 2013).

Using TurboID, we identified a new substrate of Rad53, the replication initiation factor Sld7 ( Figure 2E). Although we do not yet know the functional importance of Rad53-dependent phosphorylation of Sld7, in human cells this phosphorylation has been shown to regulate origin firing during a normal S-phase ( Ferreira *et al.*, 2021). In yeast, Rad53 inhibits origin firing in the presence of replication stress or DNA damage through phosphorylation of Sld3 and Dbf4 ( Johnson *et al.*, 2021; Lopez-Mosqueda *et al.*, 2010; Zegerman & Diffley, 2010), but it is possible that phosphorylation of Sld7 further enhances this regulation or could modulate functions of Sld7 beyond origin firing. Additionally, we identified the DNA polymerase subunit of Pol α (Pol1) as a new direct interactor with Rad53. This interaction, together with other known interactions of Rad53 with replisome proteins ( Figure 5E), is important for viability and for regulating replication progression in response to HU ( Figure 6). Therefore, from an unbiased TurboID screen, we have identified new physiologically important interactions of Rad53 with the replication machinery.

### Multiple kinases coordinate Rad53 function

Although we did not find any evidence that the checkpoint kinases directly phosphorylate Pol α, we did observe that phosphorylation of a CDK site in Pol1 mediates the interaction with Rad53 ( Figure 5). This suggests that the cell cycle is an important determinant for the targeting of Rad53 to Pol α. In addition to Mec1-dependent and Rad53-dependent phosphorylation of Mrc1 and Cdc45, respectively, to generate binding sites for Rad53, this study shows that multiple kinases control Rad53-targeting to the replisome ( Figure 6E). Such multiplicity of regulation is likely to be important to ensure that Rad53 is not aberrantly targeted to the replication machinery during a normal S-phase (in the absence of Mec1/Rad53 activation) or inappropriately recruited to replication factors outside of S-phase, for example in G1 phase, when CDK activity is low ( Figure 6E). Although a CDK site is required for the interaction between Rad53 and Pol1, our data suggests that replication stress is still important for this interaction, not only from the TurboID analysis ( Figure 2C–D), but also from the Co-IP and BiFC experiments ( Figure 3). We do not currently know why replication stress would be required for this interaction, as Pol1 is CDK phosphorylated in an unperturbed S-phase ( Figure 4B), but it may be that activation and hyperphosphorylation of Rad53 is important for this interaction *in vivo*.

### Rad53 interacts with the leading and lagging strand machinery

Studies suggest that during replication stress the checkpoint coordinates replication progression on both the leading and the lagging strand to minimise the generation of ssDNA ( He & Zhang, 2022). The mechanistic basis for this coordination is poorly understood. Rad53 binding to and subsequent phosphorylation of Mrc1, which is bound to Pol ε and the CMG helicase on the leading strand template ( Baretic *et al.*, 2020; Lou *et al.*, 2008), can slow down the CMG complex in response to stress ( McClure & Diffley, 2021). Rad53-dependent phosphorylation and binding to Cdc45 as part of the CMG complex is also important for recruiting Rad53 to replication forks ( Can *et al.*, 2019). Therefore, through both Cdc45 and Mrc1, Rad53 is actively recruited to the leading strand machinery at stalled forks ( Figure 5E). Here, we identify the Pol α catalytic subunit Pol1 as a new Rad53 interactor, suggesting that Rad53 binds not only to the leading strand through Cdc45/Mrc1, but can also bind to the lagging strand machinery ( Figure 5E).

Recruitment of Rad53 to the leading strand machinery *via* Cdc45 and Mrc1 plays an important role in regulating replication fork progression in HU ( Figure 6C–D). By itself, Pol1-dependent recruitment of Rad53 plays a minor role in replication fork progression in HU, but loss of the interaction of Rad53 with Cdc45, Mrc1 and Pol1 together leads to even greater defects in survival and fork progression ( Figure 5C–D and Figure 6C–D). This suggests that multiple interactions of Rad53 with the replication fork are important to mediate the critical functions of Rad53 in stabilising the replisome under stress. While we do not yet know the mechanistic roles of the recruitment of Rad53 by Pol α, we consider it possible that this may be required for lagging-strand specific functions of Rad53, such as PCNA unloading ( Yu *et al.*, 2014), for coordinating stalling on both strands ( He & Zhang, 2022) and/or for genomic replication independent functions of Pol α, such as telomere maintenance or double strand break repair ( Doksani & de Lange, 2014).

Significantly, a previous study has shown that Chk1, the analogous kinase to Rad53 in metazoa, can also interact with Pol α in human cells, in the presence and absence of replication stress ( Taricani *et al.*, 2009). This suggests that the checkpoint-dependent interaction with Pol α has been conserved, which may have implications for understanding how cells respond to the multitude of emerging chemotherapies that target the DNA replication checkpoint kinases ( van Bijsterveldt *et al.*, 2021).

## Data Availability

Gene Expression Omnibus: Genome-wide analysis of replication fork locations in hydroxyurea-treated Saccharomyces cerevisiae by TrAEL-seq. Accession number GSE235881, https://identifiers.org/geo:GSE235881 ( Hadjicharalambous *et al.*, 2023). Figshare: Extended data H, https://doi.org/10.6084/m9.figshare.23599356 ( Zegerman, 2023). This project contains the following extended data: Extended data Figure 1 (Viability of internally tagged Rad53 to accompany main Figure 1) Extended data Figure 2 (Validation of Rad53-TurboID to accompany main Figure 2) Extended data Figure 3 (Growth assay of split YFP tagged Rad53 and Pol1 to accompany main Figure 3) Extended data Figure 4 (CDK mediates Pol1/Pol12 phosphorylation to accompany main Figure 4) Extended data Figure 5 (Rad53 interaction with Pol1 affects fork progression to accompany main Figure 6) Extended data Table 1 (Label free quantification (LFQ) analysis of the Rad53-TurboID hits to accompany Figure 2) Extended data Table 2 (Yeast strains and antibodies used in this study) Data are available under the terms of the Creative Commons Zero "No rights reserved" data waiver (CC0 1.0 Public domain dedication).
