# Supplementary material for: Checkpoint kinase interaction with DNA polymerase alpha regulates replication progression during stress
Source: Wellcome Open Res. 2023 Jul 26;8:327. [Version 1] doi: 10.12688/wellcomeopenres.19617.1 (PMC10521137; doi:10.12688/wellcomeopenres.19617.1)
Supplement: Peer review history

# Reviewer report: Reviewer response for version 1

**DOI:** 10.21956/wellcomeopenres.21731.r64007

**Published:** 2023-09-07

**Authors:** Tim Humphrey

**Affiliations:** 1 Genome Damage and Stability Centre, Life Sciences, University of Sussex, Brighton, England, UK

Hadjicharalambous *et al.,* have sought to identify Rad53 checkpoint kinase interactors *in vivo* using TurboID, an unbiased biotin proximity-ligation approach, in *Saccharomyces cerevisiae*. In doing so the authors identify known interactors including Mrc1, Spt6, Net1, and new ones. To their credit, the authors further distinguish between substrates and interactors. In doing so they identify new substrates including the replication initiation factor Sld7, and a new direct interactor (though not a substrate), Pol alpha (Pol1), the DNA polymerase subunit responsible for priming leading and lagging strand synthesis. Interestingly, the authors show that the interaction between Rad53 and Pol1 is mediated at least in part by Pol1 T402 CDK phosphorylation, indicating multiplicity of regulation. Moreover, the authors show that Rad53 interaction with Pol1 is important for regulating replisome progression in response to replication stress. Together these findings support a role for Rad53 in modulating DNA replication through interactions with both the leading (Mrc1 and Cdc45) and lagging (Pol1) machinery.

 The manuscript is well written and structured, progressing in a clear and logical order. The data are clear and support the conclusions drawn. Experimentally, the authors combine powerful genetic and recently developed proteomic (TurboID) and sequencing technologies (TrAEL-seq) to identify and characterise new Rad53 interactions. The main focus of the manuscript is the characterization of the Rad53 interaction with Pol1, identified here, and the data are very robust. However, the functional significance of the Rad53-Pol1 interaction is less obvious. Genetically disrupting the interaction between Rad53 and the lagging strand machinery (pol1-1A) reveals only a minor effect on viability and relative growth, with no discernible effect on fork progression following HU block and release. This is despite  very strong phenotypes associated with disrupting the interaction between Rad53 and leading strand machinery alone (mrc1-AQ, cdc45-2A). Further, disrupting this interaction (pol1-1A)  together with mutations disrupting the interaction between Rad53 and the leading strand replication machinery (mrc1-AQ, cdc45-2A) reveals only a minor further impact on viability, relative growth and replication fork progression following HU block and release. Importantly, the experiments are done carefully and the results are credible. Together the findings described here represent a robust advance in our knowledge of Rad53 interactions, and support a model in which interactions between Rad53 and the replication machinery are regulated both by replication stress and the cell cycle.

Is the work clearly and accurately presented and does it cite the current literature?

Yes

If applicable, is the statistical analysis and its interpretation appropriate?

Not applicable

Are all the source data underlying the results available to ensure full reproducibility?

No source data required

Is the study design appropriate and is the work technically sound?

Yes

Are the conclusions drawn adequately supported by the results?

Yes

Are sufficient details of methods and analysis provided to allow replication by others?

Yes

Reviewer Expertise:

Genome stability; DNA repair; yeast genetics.

I confirm that I have read this submission and believe that I have an appropriate level of expertise to confirm that it is of an acceptable scientific standard.

# Reviewer report: Reviewer response for version 1

**DOI:** 10.21956/wellcomeopenres.21731.r64002

**Published:** 2023-09-07

**Authors:** Karim Labib

**Affiliations:** 1 The MRC Protein Phosphorylation and Ubiquitylation Unit, School of Life Sciences, University of Dundee, Dundee, UK

In this study, Hadjicharalambous et al use ‘TurboID’ to identify new partners and substrates of the Rad53 checkpoint kinase in budding yeast. The authors validate their approach by showing that Rad53-proximal proteins include the previously characterised partner protein Mrc1 and the previously identified substrate Sld3. The authors then focus on two Rad53-proximal proteins not previously reported. The first is the initiation factor Sld7, which the authors show is phosphorylated in a Rad53-dependent manner. The second is the Pol1 catalytic subunit of DNA polymerase alpha, which the authors show binds Rad53 in a manner dependent upon CDK-phosphorylation of Pol1, which promotes binding to the FHA1 domain of Rad53. 

 The pol1-1A allele disrupts binding to Rad53-FHA1 and increases HU sensitivity of mrc1-AQ cdc45-2A cells (defective in binding of Rad53 to previously characterised partners of Rad53). The authors suggest that binding of Rad53 to Pol1 in response to replication stress might function to target the checkpoint kinase to substrates on the lagging-strand side of replication forks. 

 Overall, the work is of high technical quality and will be of interest to those in the DNA replication field.

Is the work clearly and accurately presented and does it cite the current literature?

Yes

If applicable, is the statistical analysis and its interpretation appropriate?

Yes

Are all the source data underlying the results available to ensure full reproducibility?

Yes

Is the study design appropriate and is the work technically sound?

Yes

Are the conclusions drawn adequately supported by the results?

Yes

Are sufficient details of methods and analysis provided to allow replication by others?

Yes

Reviewer Expertise:

DNA replication and genome stability.

I confirm that I have read this submission and believe that I have an appropriate level of expertise to confirm that it is of an acceptable scientific standard.

# Reviewer report: Reviewer response for version 1

**DOI:** 10.21956/wellcomeopenres.21731.r64005

**Published:** 2023-08-22

**Authors:** Roberto Bellelli

**Affiliations:** 1 Centre for Cancer Cell and Molecular Biology, Barts Cancer Institute, Queen Mary University of London, London, England, UK

The present work from Hadjicharalambous et al. describes the identification, Bio-ID dependent, of novel direct/indirect interactors of Rad53. The authors validate a new Rad53-dependent phosphorylation of Sld7 and a novel interaction between Rad53 and Pol1, the catalytic subunit of Pola.

 They finally identify a CDK-dependent mechanism which promotes binding of Rad53 to POL1 via the Rad53 FHA1 domain and the T402 POL1 residue. Mutation of this POL1 residue significantly reduces Rad53 binding to POL1 and leads to reduced survival in condition of replication stress, particularly in combination with reduced Rad53 binding to Mrc1 and Cdc45.

 The work is technically well conducted and of interest for the DNA replication community. As such I recommend indexing. I have only a very few minor comments.

1. Figure 3: it would be good to have a representative image of YFP signal from cell untreated with HU (3C). Also, it is not clear to me why on the x scale of Fig. 3D-E-F it’s reported nuclear:cytoplasmic fluorescence ratio and not total nuclear intensity. Is it just an “internal” control of fluorescence ? If present a higher WB exposure of 3A would be also good.
2. I would perhaps modify the Title using “Fork progression during replication stress” instead of “replication progression during stress”.
3. TrailSeq shows a minor delay in fork progression (upon HU release) in the triple mutant despite significant changes in Rad53 Phosph and viability in HU (compared to single mutants).  It is possible that reduced Rad53-binding to POL1 might affect POLA activity and re-priming at stalled forks or simply POLA binding to other replication factors. This would not affect CMG activity and leading strand synthesis and as such trailseq “reads”. I was wondering if authors had checked accumulation of ssDNA (e.g. RPA) or PCNA chromatin levels in these conditions ? if not perhaps authors could discuss a bit more the various possibilities given the different location of CDC45-MRC1 (leading strand) vs POL1 (lagging etc.) and the pitfalls of TrailSeq.

Is the work clearly and accurately presented and does it cite the current literature?

Yes

If applicable, is the statistical analysis and its interpretation appropriate?

Yes

Are all the source data underlying the results available to ensure full reproducibility?

Yes

Is the study design appropriate and is the work technically sound?

Yes

Are the conclusions drawn adequately supported by the results?

Yes

Are sufficient details of methods and analysis provided to allow replication by others?

Yes

Reviewer Expertise:

Genome stability; cancer biology

I confirm that I have read this submission and believe that I have an appropriate level of expertise to confirm that it is of an acceptable scientific standard.
